# Antioxidant and antihyperlipidemic effects of hawthorn extract (*Crataegus oxyacantha*) in broiler chickens

**DOI:** 10.1002/vms3.1414

**Published:** 2024-03-20

**Authors:** Behnam Ahmadipour, Majid Kalantar, Samira Abaszadeh, Hossein Hassanpour

**Affiliations:** ^1^ Department of Animal Science Faculty of Agriculture Shahrekord University Shahrekord Iran; ^2^ Department of Animal Science Qom's Agricultural Research and Education Center Agricultural Research, Education, and Extension Organization, Jihad‐e‐Keshavarzi Ministry Qom Iran; ^3^ Department of Basic Sciences Faculty of Veterinary Medicine Shahrekord University Shahrekord Iran

**Keywords:** broilers, gene expression, hawthorn extract, lipid profile, oxidative stress

## Abstract

**Background:**

One of the main problems in the poultry industry is excess body fat, and the anti‐fat effect of *Cratagus* extract has been confirmed in several studies.

**Objectives:**

The present study was carried out to investigate the effects of hawthorn extract (*Crataegus oxyacantha*) on growth performance, haematological variables and hepatic gene expression in broiler chickens reared at high altitude (2100 m).

**Methods:**

A total of 225‐day‐old chicks (Ross 308) were randomly distributed into three treatments. Experimental treatments were prepared by adding 0.0, 0.2 and 0.4 mL of hawthorn extract per litre of consumption of water.

**Results:**

The results showed that weight gain and feed conversion ratio were significantly improved and abdominal fat decreased by consumption of two levels of *Crateagus* extract when compared to the control (*p *< 0.05). Consumption of hawthorn extract decreased circulatory levels of malondialdehyde, triacylglycerol, total cholesterol and low‐density lipoproteins cholesterol but increased ferric reducing antioxidant power and high‐density lipoproteins cholesterol (*p* < 0.05). Hawthorn extract caused an up‐regulation of catalase, superoxide dismutase1, glutathione peroxidase1 and peroxisome proliferator‐activated receptor alpha but reduced the expression of key lipogenic enzymes (*p *< 0.05).

**Conclusions:**

Overall, consumption of 0.4 mL hawthorn extract per litre of drinking water, improved growth performance, suppressed lipogenesis and enhanced antioxidant response.

## Graphical abstract

Graphical Abstract: Hawthorn extract (*Crataegus oxyacantha*) has antioxidant and hyperlipidemic properties. The use of this extract in broilers improves weight gain and feed conversion ratio. Hawthorn extract caused an up‐regulation of catalase, superoxide dismutase1, glutathione peroxidase 1 and peroxisome proliferator–activated receptor alpha but reduced the expression of key lipogenic enzymes. Drinking hawthorn extract decreased circulatory levels of malondialdehyde, triacylglycerol, total cholesterol and low‐density lipoproteins cholesterol but increased ferric reducing antioxidant power and high‐density lipoproteins cholesterol.

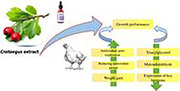

## 1 INTRODUCTION

Modern broiler chickens are characterized by an increased growth rate through genetic selection. Increase in growth rate is associated with fat tissue accumulation as body fat deposition (Ahmadipour et al., 2018, 2017). Unwanted abdominal and subcutaneous fat depositions are the main sources of waste by industry or consumers (Wood et al., 2008; Wang et al., 2017). One of the main problems in the poultry industry is excessive body fat due to increased incidence of metabolic diseases and physiological disorders which lead to an increase in mortality (Ahmadipour et al., 2018; Wang et al., 2017). In addition, the consumer's reluctance to consume excess fat should be considered (Sanz et al., 2000; Wang et al., 2017).

The excess fat in broiler chicken in the form of abdominal fat is the chief portion of body fat which constitutes approximately 2%–3% of the bird's live weight (Fouad & El‐Senousey, 2014; Sanz et al., 2000). This percentage varies depending on several factors affecting fat deposition in broiler chickens, including genetics, nutrition, sex and age of the broiler chicken (Havenstein et al., 2003; Sanz et al., 2000). Physiologically, excess fat in broiler chickens is not essential for the body's vital functions, but because most fats are unsaturated and prone to oxidation, they impose a heavy burden on the body's antioxidant system (Sanz et al., 2000; Wood et al., 2008). Chickens have a higher content of polyunsaturated fatty acids in their adipose tissue than other animals, such as pigs, cattle and sheep, and therefore have a greater tendency to oxidize adipose tissue (Wang et al., 2017; Wood et al., 2008). So, lipid oxidation in poultry meat is considered to be the main cause of quality damage related to the flavour, colour, taste and nutritional composition of meat and poultry products (Sanz et al., 2000; Wang et al., 2017). Control of poultry diets with methods such as adjusting the ratio of energy to protein, reducing dietary fat content, adopt nutritional strategies, including feed restriction and the use of medicinal plant products, are related fields of research (Attia et al., 2017; Wang et al., 2017).

Hawthorn (*Crataegus* Sp.), also known as thornapple, maytree or hawberry, is a flowering shrub of the rose family. Common species include *Crataegus monogyna*, *Crataegus laevigata* and *Crataegus oxyacantha*, which is cultivated in various countries of the world. *C. oxyacantha* is widely distributed in different parts of Asia (Barros et al., 2011; Chang et al., 2002; Long et al., 2006). The hawthorn leaves, berries and flowers are used as herbal drugs. They contain chemicals called flavonoids, which have multiple therapeutic effects (Dahmer & Scott, 2010; Yang & Liu, 2012). *C. oxyacantha* is one of the most famous species of the hawthorn family that is used in traditional medicine due to its antioxidant and antihyperlipidemic effects (Ahmadipour et al., 2017, Ahmadipour, Kalantar, Kalantar, et al., 2019, Ahmadipour, Kalantar, Hosseini, et al., 2019; Long et al., 2006; Yang & Liu, 2012).

The results of the research indicated that several beneficial results of hawthorn extract were determined, including (1) lowering body and liver weight and liver to‐body weight ratio; (2) improving serum parameters and liver dysfunction; (3) decreasing hepatic lipid accumulation; (4) increasing circulating adiponectin levels and up‐regulating the expression of adiponectin receptors in the liver; and (5) activating AMP‐activated protein kinase (AMPK) and altering AMPK‐mediated SREBP‐1c, peroxisome proliferator‐activated receptor alpha (PPAR‐α) and their downstream targets (Li et al., 2015).

Lipid‐lowering effects of hawthorn have been widely studied in many studies using different animals. Plant chemicals demonstrated the ability to lower triacylglycerol (TG), total cholesterol (TC), low‐density lipoproteins cholesterol (LDL) and very‐low‐density lipoproteins cholesterol and to increase high‐density lipoproteins cholesterol (HDL) in plasma, and these effects have been explained by several possible mechanisms (Wu et al., 2020; Yang & Liu, 2012). The main chemical constituents of hawthorn flavonoid extract include flavonoids (1%–2%), oligomeric proanthocyanidins (1%–3%) and other bioactive components (e.g. triterpene acids, organic acids, sterols and cardioactive amines) (Ahmadipour et al., 2020). These compounds are reported to have many pharmacological effects, including neuroprotective, hepatoprotective, cardioprotective and also antioxidant and antihyperlipidemic effects (Ahmadipour et al., 2020; Barros et al., 2011; Chang et al., 2002; Kao et al., 2005; Kirakosyan et al., 2003; Liu et al., 2011; Long et al., 2006). Hawthorn plant contains 7 mg/g of protein, 2 mg/g of fat, 30–40 mg/g of pectin, 30–50 mg/g of organic acids, 3–8 mg/g of tannins, 0.5–1.5 mg/g of amino acids, 0.89 mg/g of vitamin C, 0.89 mg/g of vitamin D and 0.65 mg/g of flavonoids. A total of 65 chemical components were identified in hawthorn essential oil, mainly eicosanoids (12%–17%), 21 alkanes (11%–16%), linalool (6%–11%), hexadecanoic acid (1%–11%) and 19 alkanes (3%–7%) (Chang et al., 2002; Liu et al., 2011; Zhang et al., 2022).

Flavonoids and triterpenoids as two major constituents of hawthorn have anti‐inflammatory and anti‐hyperlipidemic properties. Part of the mechanism for the anti‐hyperlipidemic effects of hawthorn fruit might also involve the direct protection of human LDL from oxidation (Kao et al., 2005; Kirakosyan et al., 2003; Wu et al., 2020).

There is not enough information about the antioxidant and antihyperlipidemic effects of hawthorn in broiler chickens. According to the facts that some compounds in hawthorn extract have strong antioxidant potential and some of its compounds have lowering plasma lipids effects, the objectives of the present study were to examine the effects of different consumption of levels of hawthorn flavonoid extract to prevent oxidation and hyperlipidemic of broiler chickens. To the best of our knowledge, there has been no report on the effect of hawthorn flavonoid extract in the mentioned fields.

## 2 MATERIALS AND METHODS

### 2.1 Gas chromatography/mass spectrometry (GC/MS) procedure

The bioactive molecules in hawthorn extracts were determined using a gas chromatograph attached to a mass spectrometer and displayed in Table 1. The HP‐5MS 5% phenylmethyl siloxane capillary column (30.00 m 0.25 mm, 0.25 m film thickness) with an Agilent 7890A gas chromatograph (Agilent Technologies) was used to evaluate Crataegus‐Drop. The temperature was initially connected to the stove at 60°C for 4 min, then increased by 4°C per minute until it reached 260°C. The detector and injector had respective temperatures of 300 and 290°C. The carrier gas streamed at a rate of 2 mL/min and was helium. In the split mode, samples (1 µL) were manually injected. Peak area percentages were used to produce the numerical data. A mass‐specific detector (Agilent Technologies) was coupled to the gas chromatograph. The ionization voltage, 70 eV, and ion source temperature, 200°C, were among the EI‐MS working parameters. A similar arrangement of *n*‐alkanes (C5–C24) was used to analyse maintenance records for all components, while maintaining the same environmental conditions as the samples. By comparing mass spectral fragmentation designs and maintenance durations (using the WILLEY/ChemStation Information System), oil components were identified (Adams, 2007).

**TABLE 1:** Composition of hawthorn extract (*Crataegus oxyacantha*).

Compound	Concentrat (%)
Formic acid	2.10
2‐Formylhistamine	0.43
Catechol	12.85
Sorbitol	8.75
Acetic acid	1.91
γ‐Sitosterol	3.22
*n*‐Hexadecanoic acid	3.63
Erythritol	2.11
5‐Hydroxymethylfurfural	27.06
Isosorbide	1.07
Ethyl 3‐methylbut‐3‐enyl carbonate	2.96
3‐Ethyl‐3‐heptanol	2.82
Catechin	10.64
Hydroquinone	2.53
Ethanol	3.62
Propargyl alcohol	3.97
1,3‐Butadiene‐1‐carboxylic acid	1.46
1‐Bromoeicosane	0.92
3‐Furanmethanol	1.74
5‐Methyl‐1‐nitropyrazole	2.21

### 2.2 Determination of total phenol content

The total phenolic content of the samples was determined using the Folin–Ciocalteu method described by Singleton et al. (1999). Briefly, 50 µL of Crataegus‐Drop (concentration 1 mg/mL in methanol) was mixed with 3.95 mL of distilled water and 250 µL of Folin–Ciocalteu, respectively. Results are presented in Table 2 as mg gallic acid equivalents/g lyophilized extract (mg GAE/g E).

**TABLE 2:** Total phenolic, flavonoid content and antioxidant activity for hawthorn extract.

Sample	TPC (mg GAE/g extract)	TFC (mg QE/g extract)	DPPH (% inhibition)
Hawthorn extract	102.43 ± 2	12.11 ± 0.5	91.80 ± 0.5

Abbreviations: DPPH, 1,1‐diphenyl‐2‐picrylhydrazyl; TPC, total phenolic compounds; TFC, total flavonoid compounds.

### 2.3 Measurement of total flavonoid content

Total flavonoid content (TCF) was determined according to the method described by Cottica (2015). Briefly, 0.25 mL of 5% aluminium chloride (ethanol) was mixed with 0.5 mL of Crataegus‐Drop (1 mg/mL concentration in methanol). Total flavonoids are expressed in Table 2 as mg quercetin equivalent/g lyophilized extract (mg QE/g 128 E).

### 2.4 Antioxidant evaluation (DPPH free radical scavenging assay)

The antioxidant capacity of Crataegus‐Drop was measured according to the method of Mensor et al. (2001) using 1,1‐diphenyl‐2‐picrylhydrazyl (DPPH). A volume of 200 µL of Crataegus‐Drop (concentration 1 mg/mL in methanol) was added to 80 µL of 0.3 mM methanolic DPPH solution. DPPH was expressed as the inhibition rate (%) of the lyophilized extract in Table 2.

### 2.5 Birds and experimental facility

The experiment was conducted in the experimental facility of Shahrekord University, Shahrekord, Iran. A total of 225‐day‐old broilers (Ross 308) were randomized across 15‐floor pens. Each pen measured 2 m^2^ (15 birds per pen) and was equipped with a bell drinker and a feed trough. All chicks were kept, maintained and treated according to the accepted standards for the human treatment of animals. One‐day‐old chicks were then allocated to pens (15 birds per pen with a weighted 47.8 g on average) so that all pens had equal initial body weights (717 ± 10 g). For all experimental groups, a basal diet of the corn‐soybean meal was formulated for starting (1–3 weeks of age) and growing (3–6 weeks of age) periods according to National Research Council (NRC) (1994) recommendations (Table 3).

**TABLE 3:** Ingredients and chemial composition of experimental diets (1–42 days).

Item (% unless noted)	Starter (1–10 days)	Grower (11–21 days)	Finisher (22–42 days)
Corn	53.77	60.58	66.71
Soybean meal (44% CP)	36.97	29.78	28.10
Corn gluten meal	3.48	4.60	0.00
Soy oil	1.35	1.00	1.6
Dicalcium phosphate	1.52	1.45	1.2
Oyster shell	1.32	1.14	1.1
Salt	0.24	0.25	0.28
NaHCO3	0.20	0.20	0.15
dl‐Methionine	0.31	0.20	0.19
l‐Lysine	0.28	0.26	0.16
l‐Threonine	0.06	0.04	0.01
Mineral supplement^a^	0.25	0.25	0.25
Vitamin supplement^b^	0.25	0.25	0.25

Calculated composition			
Metabolizable energy (kcal/kg)	2910	3050	3120
Crude protein (%)	22.74	20.90	18.10
Digestible methionine (%)	0.63	0.52	0.43
Methionine + cystine	0.91	0.82	0.69
Digestible lysine (%)	1.25	1.10	0.97
Digestible threonine (%	0.80	0.70	0.60
Calcium (%)	1.05	0.95	0.87
Available P (%)	0.50	0.48	0.43
DCAB (mEq/kg)	241	216	207

Abbreviation: DCAB, dietary cation‐anion balance.

^a^ Provided the following per kg of diet: vitamin A (trans retinyl acetate), 3600 IU; vitamin D3 (cholecalciferol), 800 IU; vitamin E (dl‐α‐tocopheryl acetate), 7.2 mg; vitamin K3, 1.6 mg; thiamine, 0.72 mg; riboflavin, 3.3 mg; niacin, 0.4 mg; pyridoxin, 1.2 mg; cobalamine, 0.6 mg; folicacid, 0.5 mg; choline chloride, 200 mg.

^b^ Provided the following per kg of diet: Mn (from MnSO_4_·H_2_O), 40 mg; Zn (from ZnO), 40 mg; Fe (from FeSO_4_·7H_2_O), 20 mg; Cu (from CuSO_4_·5H_2_O), 4 mg; I [from Ca (IO_3_)2·H_2_O], 0.64 mg; Se (from sodium selenite), 0.08 mg.

Experimental treatments were prepared by adding 0.0, 0.2 and 0.4 mL of HFE (HE 00152, Crataegus‐Drop 6260) per litre of consumption of water (measured pH = 7.05 and total dissolved solids = 2000 ppm). The HFE contained 0.25 and 0.50 mg/L of total flavonoid compounds. In this way, birds in 0.1 and 0.2‐mL HFE groups received 0.05–0.10 mg of total flavonoid compounds daily. *C. oxyacantha* (common hawthorn) is an endemic member of the Rosaceae family that grows in Europe, Africa and Asia, where it is commonly found as a shrub or small tree 5–10 m tall (Chang et al., 2002). Hawthorn as a traditional medicinal plant is a plant locally called Sorkh‐e‐valik or Zalzalak that is found in the western and central regions of Iran (Salehi et al., 2009). Oral Crataegus‐Drop is a well‐known herbal drug therapy that is available at the pharmacy under an approved code and supervision of the Iranian Food and Drug Administration (HE 00152, Crataegus‐Drop 6260). The flavonoid extract of this product contains biologically active flavonoid compounds (polyphenols) such as anthocyanidins and proanthocyanidins (also known as bioflavones or procyanidins). Each millilitre of oral Crataegus‐Drop 6260 contained 2.5 mg of total flavonoid compounds in the form of hyperoside (21.4% polyphenols and 19.7% procyanidins), produced by Iran Darouk Pharmacy Co, under the production code of 3067‐88‐02. The determination of total phenolic compounds in Crataegus‐Drop 6260 was carried out through the colorimetric method in accordance with the standard extraction procedure of the manufacturer (Ahmadipour, 2018).

### 2.6 Measurements

Body weight gain and feed intake were recorded in 1–20, 21–40 and 1–40 days of age periods. The feed conversion ratio (FCR) was also calculated for the same periods. At 40 days, 10 birds were selected from each group for blood collection and processing. The selected birds had body weights within ∼5% of the average pen body weight. Blood samples (∼3 mL) were collected from the brachial vein and centrifuged at 2500 × *g* for 10 min to separate sera. Serum samples were tested for TC, TG, HDL, LDL and malondialdehyde (MDA). TC, TG, HDL and LDL were measured using commercial laboratory kits following the manufacturer's manuals (Pars Azmoon). MDA, as a biomarker of lipid peroxidation, was assayed according to Nair and Turner (1984). Ferric reducing antioxidant power (FRAP) assay was measured according to the procedure described by Sudha et al. (2012) with some modifications. The FRAP reagent contained 2.5 mL of a 10 mM TPTZ (2,4,6‐tripyridyl‐*s*‐triazine) solution in 40 mM HCl, 2.5 mL of 10 mM FeCl_3_∙6H_2_O and 25 mL of 300 mM acetate buffer (pH 3.6). Birds were then euthanised by CO_2_ to determine carcass variables, including weights of breast, thigh, liver and abdominal fat according to Ahmadipour, Kalantar, Hassanpour, et al. (2019), Ahmadipour, Kalantar, Hosseini, et al. (2019).

### 2.7 Quantitative real‐time PCR analysis

The liver of 10 birds per treatment was harvested, homogenized and treated by a digestion buffer (RNX‐Plus reagent; Sinaclon Bioscience). An aliquot of 200 µL chloroform was added to the digested samples and centrifuged at 16,000 × *g* for 15 min (under a cool temperature, 4°C). The supernatant was taken to precipitate RNA using the equal volume of isopropanol. The samples were then rinsed with 75% ethanol, centrifuged and the resulting pellet dissolved in the appropriate amount of diethyl dicarbonate‐treated water. The extracted RNA was treated with a DNAase kit (Sinaclon Bioscience) to remove residual DNA. The quantity of RNA was checked by O.D. (A260/280) using a biophotometer (Eppendorf). RNA with an absorbance ratio between 1.8 and 2.2 was used for the synthesis of cDNA.

Total RNA was converted to cDNA by means of the PrimeScript RT Reagent Kit (Takara Bio Inc.). The levels of superoxide dismutase 1 (SOD1), catalase (CAT), Glutathione peroxidase1 (GPX1), PPARα and tyrosine 3‐monooxygenase/tryptophan 5‐monooxygenase activation protein zeta (YWHAZ) transcripts were determined by real‐time RT‐PCR using SYBR Premix Ex Taq II kit (TliRnase H Plus; Takara Bio Inc.). YWHAZ was used as an endogenous reference gene to normalize the input load of cDNA across samples (Hassanpour et al., 2018). Table 4 represents the specific primers of PCR, designed with the online software of Primer‐Blast (https://www.ncbi.nlm.nih.gov/tools/primer‐blast/index.cgi?LINK_LOC = BlastHome). The amplifications were run in a real‐time PCR cycler (Rotor‐Gene Q 6000; Qiagen) in three replicates per liver sample. Data were analysed using LinRegPCR software version 2012.0 (Amsterdam) to provide the threshold cycle number and reaction efficiency (Ruijter et al., 2009). Relative transcript levels (gene/YWHAZ) were calculated using the efficiency‐adjusted Paffl methodology (Dorak, 2006; Hassanpour et al., 2018).

**TABLE 4:** Details of the primers used for quantitative real‐time PCR analysis of chicken mRNAs.

Target	Target Primers	PCR product (bp)	Accession no.
YWHAZ	F:5′‐AGGAGCCGAGCTGTCCAATG‐3′	84	NM_001031343.1
	R:5′‐CTCCAAGATGACCTACGGGCTC‐3′		
PPAR*α*	F:5‐GCTGTGGAGATCGTCCTGGT‐3	163	NM_001001464.1
	R:5‐GTGACAAGTTGCCGGAGGTC‐3		
CPT1A	F:5'‐ACTCTTCTCGGGACGGAAGC‐3'	113	NM_001012898
	R:5'‐GTGGCCGGACTGATTCCAGA‐3'		
FAS	F:5'‐GCTGGCTACAGTGGTGGACT‐3'	129	NM_205155.2
	R:5'‐CCACCTCGAACCACCAAAGC‐3'		
LPL	F:5'‐CTCCGATCCCGAAGCTGAGATG‐3'	106	NM_205282.1
	R:5'‐CTGTCCAGGAACCAGGTAGC‐3'		
ACAC	F:5'‐CAACGAGTCGGGCTACTACC‐3'	146	J03541.1
	R:5'‐GGTCCTTGGTCACGTATGGG‐3'		
MAE	F:5'‐GGACCTCGAAGCCTTCATCC‐3'	77	AF408407.1
	R:5'‐GCCAGGTGTATGAGTCAGCC‐3'		
CAT	F:5′‐TGGCGGTAGGAGTCTGGTCT‐3′	112	NM_001031215.1
	R:5′‐GTCCCGTCCGTCAGCCATTT‐3′		
SOD1	F:5′‐CACTGCATCATTGGCCGTACCA‐3′	223	NM_205064.1
	R:5′‐GCTTGCACACGGAAGAGCAAGT‐3′		
GPX1	F:5′‐GCTGTTCGCCTTCCTGAGAG‐3′	118	NM_001277853.1
	R:5′‐GTTCCAGGAGACGTCGTTGC‐3′		

Abbreviations: ACAC, acetyl‐coA carboxylase; bp, base pair; CAT, catalase; CPT1A, Carnitine palmitoyltransferase 1A; FAS, fatty acid synthase; GPX1, glutathione peroxidase 1; LPL, lipoprotein lipase; MAE, malic enzyme; PPAR*α*, peroxisome proliferator activated receptor alpha; SOD1, superoxide dismutase 1.

### 2.8 Statistical analysis

The ANOVA procedure of SAS Institute Inc (2007) software was adopted to analyse the data in a completely randomized design. Duncan's test compared significant differences among the means, and the level of probability less than 0.05 was considered statistically different. Linear and quadratic effects of *Crateagus* extract levels were studied using orthogonal polynomial contrasts to compare with the control group.

## 3 RESULTS

The effects of consumption of treatments on the growth performance of broiler chickens are presented in Table 5. Broilers’ weight gain and FCR were significantly improved by adding 0.2 and 0.4 mL of hawthorn extract per litre of consumption of water when compared to the control (*p *< 0.05).

**TABLE 5:** Effects of *Crateagus* extract (*C*E) on broiler's growth performance.

Drinking levels of *Crateagus* extract (mL/L)					*p*‐Value		
	Control (0)	0.2	0.4	SEM	control vs. *C*E	Linear^1^	Quadratic
Feed intake (g/bird)							
1–42 days of age	4162.7	4226.6	4161.1	48.10	0.5680	0.6096	0.3616
Weight gain (g/bird)							
1–42 days of age	2187.6^b^	2376.3^a^	2428.0^a^	20.20	0.0001	0.0001	0.1049
Feed conversion ratio							
1–42 days of age	1.90^a^	1.78^b^	1.71^b^	0.008	0.0001	0.0001	0.0006

*Note*: Each mean represents values from five replicates. Means in the same raw with different letters are significantly different (*p* < 0.05).

^1^ Orthogonal contrast.

Data for carcass characteristics are presented in Table 6. Carcass, breast and thigh yield were significantly increased by adding 0.4 mL of hawthorn extract per litre of consumption of water when compared to the control (*p *< 0.05). The proportions of liver and abdominal fat to live body weight were linearly reduced by the consumption of hawthorn extract, and there was significant difference between the control and hawthorn extract at 0.2 and 0.4 mL (*p *< 0.05).

**TABLE 6:** Effect of *Crateagus extract* on carcass variables in broiler chickens measured at 42 days of age.

Drinking levels of Crateagus extract (mL/L)					*p*‐Value		
(as % BW)	Control (0)	0.2	0.4	SEM	control vs. *C*E	Linear^1^	Quadratic
Carcass	69.60^b^	70.20^ab^	71.20^a^	0.430	0.0480	0.0529	0.1173
Breast	30.60^b^	31.10^ab^	31.40^a^	0.240	0.1053	0.0493	0.4233
Thigh	28.30^b^	29.00^ab^	29.80^a^	0.270	0.0026	0.0023	0.0599
Liver	2.99^a^	2.67^b^	2.37^c^	0.310	0.0010	0.0008	0.0490
Abdominal fat	1.50^a^	1.38^b^	1.21^c^	0.090	0.1055	0.0815	0.2106

*Note*: Superscripts in the same row with different letters are significantly different (*p* < 0.05). Each mean represents values from 10 replicates.

^1^ Orthogonal contrast.

Table 7 depicts serum and blood variables in broilers receiving 0.2 and 0.4 mL of hawthorn extract per litre of consumption of water. Regression analysis indicated a significant linear response for serum variables when *Crateagus* extract consumption. Two‐steps treatments increased serum levels of FRAP (µmol/L) (*p *< 0.05) and decreased serum triglycerides and LDL levels (*p* < 0.05). Adding 0.4 mL of hawthorn extract caused a significant reduction in TC and serum MDA level while increasing HDL compared to the group that received 0.4 mL HFE and the control group (*p* < 0.05). Orthogonal contrast between the control vs. *Crateagus* extract groups was significant for serum variables.

**TABLE 7:** Effect of *Crataegus* extract (*C*E) on serum and blood variables in broiler chickens measured at 42 days of age.

Drinking levels of crateagus extract (mL/L)					*p*‐Value		
Metabolites	Control (0)	0.2	0.4	SEM	control vs. *C*E	Linear^1^	Quadratic
Malonedialdehyde (µmol/L)	2.48^a^	2.16^ab^	1.88^b^	0.162	0.0521	0.0310	0.2376
FRAP (µmol/L)	3.65^c^	4.28^b^	5.58^a^	0.178	0.0001	0.0001	0.0001
Triglycerides (mg/dL)	81.70^a^	72.70^b^	64.60^c^	2.112	0.0001	0.0001	0.0129
Cholesterol (mg/dL)	116.20^a^	108.10^a^	96.00^b^	2.952	0.0004	0.0008	0.0085
HDL (mg/dL)	63.90^b^	71.70^ab^	78.40^a^	3.702	0.0381	0.0227	0.2168
LDL (mg/dL)	42.90^a^	39.20^b^	35.30^c^	0.939	0.0001	0.0001	0.0074

*Note*: Each mean represents values from 10 replicates. Superscripts in the same row with different letters are significantly different (*p* < 0.05).

Abbreviations: FRAP, ferric reducing antioxidant power; HDL, high‐density lipoproteins cholesterol; LDL, low‐density lipoproteins cholesterol.

^1^ Orthogonal contrast.

Table 8 depicts the effects of hawthorn extract on the expression of antioxidant genes in the liver of broiler chickens. The results describe that antioxidant genes (SOD1, GPX1 and CAT) had up‐regulation under the effects of using 0.4 mL of hawthorn extract.

**TABLE 8:** Effect of *Crataegus* extract (*C*E) on antioxidant gene expression in the liver of broiler chickens measured at 42 days of age.

Drinking levels of Crateagus extract (mL/L)					*p‐*Value		
Gene	Control (0)	0.2	0.4	SEM	control vs. *C*E	Linear^1^	quadratic
CAT	0.0071^b^	0.0961^b^	0.3608^a^	0.060	0.0013	0.0069	0.0054
GPX	0.0028^b^	0.0163^b^	0.0952^a^	0.016	0.0016	0.0171	0.0031
SOD1	0.0103^b^	0.0454^a^	0.0603^a^	0.008	0.0019	0.0007	0.2394

*Note*: Superscripts in the same row with different letters are significantly different (*p* < 0.05). Each mean represents values from 10 replicates.

Abbbreviations: CAT, catalase; GPX1, glutathione peroxidase; SOD1, superoxide dismutase 1.

^1^ Orthogonal contrast.

Table 9 represents the effects of hawthorn extract on the expression of lipogenic genes in the liver of broiler chickens. The results showed that genes involved in lipid metabolism were significantly down‐regulated by the consumption of hawthorn extract. Orthogonal contrasts of the expression fatty acid synthase, acetyl CoA carboxylase, lipoprotein lipase and malic enzyme between the control vs. *Crateagus* extract groups were significant. This evidence is that *Crateagus* extract consumption significantly prevents expression of genes in a linear manner.

**TABLE 9:** Effect of *Crataegus* extract (*C*E) on lipogenic gene expression in the liver of broiler chickens measured at 42 days of age.

Drinking levels of *Crateagus* extract (mL/L)					*p‐*Value		
Gene	Control (0)	0.2	0.4	SEM	control vs. *C*E	Linear^1^	quadratic
PPAR*α*	0.0082^b^	0.0189^b^	0.2978^a^	0.048	0.0004	0.0197	0.0006
CPT1A	0.0348^a^	0.0075^b^	0.0001^b^	0.005	0.0014	0.0096	0.0043
Fatty acid synthase	0.0709^a^	0.0387^ab^	0.0198^b^	0.012	0.034	0.0159	0.3143
Acetyl CoA carboxylase	0.0247^a^	0.0111^b^	0.0028^b^	0.003	0.0051	0.0028	0.1832
Lipoprotein lipase	0.0119^a^	0.0052^ab^	0.0016^b^	0.002	0.0201	0.0087	0.3064
Malic enzyme	0.0287^a^	0.0077^b^	0.0009^b^	0.005	0.0042	0.0014	0.3842

*Note*: Superscripts in the same row with different letters are significantly different (*p* < 0.05).

Abbreviations: CPT1A, Carnitine palmitoyltransferase 1A; FAS, fatty acid synthase; LPL, Each mean represents values from 10 replicates; PPAR*α*, peroxisome proliferator activated receptor alpha.

^1^ Orthogonal contrast.

## 4 DISCUSSION

The effects of hawthorn on antioxidant and antihyperlipidemic conditions of broiler chickens demonstrate that treatment with hawthorn in a dose of 0.4 mL/L of consumption of water results in a significant reduction in TC and LDL and a significant increase in HDL. Hawthorn extract has the ability to demonstrate a lipid‐lowering effect in animal models of hyperlipidemia (Kausar et al., 2011; Kwok et al., 2013; Yoo et al., 2016). Hawthorn treated with *n*‐ethanol and ethyl acetate alleviated hyperlipidemia by regulating disorders of lipid, energy and amino acid metabolism and reducing oxidative stress, resulting in a decrease in plasma levels of TC, triglycerides and low‐density lipoprotein cholesterol (LDL‐C) in hyperlipidemic rats (Kausar et al., 2011; Zhang et al., 2022). Similarly, hawthorn extract was able to improve hyperlipidemia by improving the lipid profile, reducing oxidative stress and lowering total and LDL cholesterol levels in the serum of ovariectomized rats (Yoo et al., 2016). Moreover, the alcoholic extract of Zhongtian hawthorn promotes cholesterol elimination (Hu et al., 2016). Several previous preclinical studies reported that the hawthorn extract reduces concentrations of TC as well as LDL, and TG in different laboratory animals as well as humans with hyperlipidemia (Tassell et al., 2010; Venskutonis, 2018; Wu et al., 2020). The results of Table 7 in this experiment also prove that hawthorn extract had a serum lipid‐lowering effect on broiler chickens. Moreover, hawthorn extract also causes a significant decrease in lipid deposits in rabbit liver and aorta (Verma et al., 2007). It has been found that reductions in TC in hamsters fed a high‐fat diet following treatment with hawthorn were accompanied by an increased expression of the liver enzyme cholesterol hydroxylase (the rate‐limiting enzyme in the synthesis of bile acid from cholesterol) and lower levels of intestinal acyl‐CoA: cholesterol acyltransferase (an enzyme involved in cholesterol uptake and absorption by the intestine), resulting in decreased blood lipid concentrations (Zhang et al., 2002). Furthermore, the treatment of the human hepatocyte cell line with hawthorn extract induced a 5.6‐fold up‐regulation of LDL‐receptor transcription in addition to downregulated ApoB (ApoB is a structural component of serum LDL particles) synthesis in a concentration‐dependent manner (Xu et al., 2009). These in turn modulate both lipogenesis and lipolysis, resulting in reduced blood lipid concentrations. The changes in lipid parameters observed in our study are in accordance with the previous preclinical studies that demonstrate the antihyperlipidemic effect of hawthorn extract in animal models of hyperlipidemia (Hu et al., 2016; Verma et al., 2007; Xu et al., 2009; Zhang et al., 2022).

Phenolic compounds, flavonoids and triterpenoids are important components of natural antioxidant substances (Venskutonis 2018). Hawthorn polyphenol extract can regulate the apoptosis of HaCaT cells by reducing reactive oxygen species production, DNA damage and p53 activation, effectively reducing UVB‐induced photodynamic and non‐photodynamic damage and providing protection to the skin (Liu et al., 2019; Zhang et al., 2022). Hawthorn total flavonoid extract and (−)‐epicatechin inhibited acute cellular oxidative stress triggered by the free radical initiator Aminobenzyl phsphonic acid (ABPA). Both total flavonoid extract and (−)‐epicatechin at all concentrations tested were taken up by cells and both significantly inhibited the oxidation of DCFH (Zhang et al., 2022). The report of Wang et al. (2011) indicated that flavonoid fraction showed inhibitory effects on TG and glucose absorption and accelerating effects on gastrointestinal transit in vivo and suppressed the accumulation of TG and free fatty acid. It also suppressed the gene expressions of C/EBP𝛼, PPAR𝛾, SREBP 1c, aP2 and adiponectin in vitro. As we know, LPL plays an important role in lipoprotein metabolism and is expressed in various tissues, especially adipose and muscle tissue, where it plays different roles (Wang et al., 2017; Wood et al., 2008). Hawthorn flavonoids increase LPL expression through a PPAR𝛾‐dependent mechanism directed towards the identification of the components (Fan et al., 2006). Along with our study, Akila and Devaraj (2008) showed that hawthorn extract significantly restored the activity of antioxidant enzymes such as superoxide dismutase, CAT, GPX and glutathione, thereby restoring the antioxidant status of the organism to almost normal levels.

Alcoholic extract of *C. oxyacantha* (AEC) pretreatment maintained mitochondrial antioxidant status and prevented mitochondrial lipid per‐oxidative damage and decrease in Krebs cycle enzymes induced by isoproterenol in rat heart (Wang et al., 2011, 2013). AEC can act on myocardial tissue to reduce iNOS expression and downregulate COX‐2 to exert anti‐inflammatory effects (Wang et al., 2013). Hawthorn flavonoids exert antihyperlipidemic effects by acting on the PPARγ pathway to increase LDL expression in blood vessels (Fan et al., 2006).

According to a report by Venskutonis (2018), methanol and ethanol extracts of *Crataegus azarolus* exerted substantial antioxidant, anti‐inflammatory and antiproliferative capacities, which were evaluated by measuring the secreted amounts of the proinflammatory mediator prostaglandin E2, and by assaying the mRNA levels of the proinflammatory cytokines (IL‐α, IL‐β and Il‐6), chemokines (CCL3 and CCL4) and inflammation‐sensitive COX2 and iNOS enzymes.

In agreement with our data, Yoo et al. (2016) also confirmed that hawthorn extract could improve body performance, lipid profile, potentiate enzymatic antioxidants and modulate oxidative stress in rat.

In the study of Pirany et al. (2020) differential expression of genes implicated in liver lipid metabolism in broiler chickens differing in weight was investigated. Lipid parameters and expression of ACACA, APOA1, CPT1A, FASN, FOXO1, LIPG, PPARα and SIRT1 genes involved in lipid metabolism were investigated in two groups of high (HW) and low (LW) weight broilers from the same strain. In this regard, according to the results, blood cholesterol and liver triglyceride levels were significantly increased in HW chickens compared to LW broilers, whereas other parameters, that is blood triglyceride, blood HDL/LDL, liver cholesterol and total liver fat showed no significant changes in either group. Moreover, the relative expression of ACACA, APOA1 and CPT1A genes was significantly lower in the liver tissues of HW broilers than in the LW group. The mRNA levels of these three genes showed a significant negative correlation with abdominal fat deposition and the live weight of broilers. However, relative expression of FASN, FOXO1, LIPG, PPARα and SIRT1 hepatic genes did not differ among broilers. So it was concluded that of eight hepatic genes implicated in lipid metabolism, only the expression of three (ACACA, APOA1 and CPT1A) was significant for fat and leanness within the same strain of chicken. As reducing body fat is a major goal in the broiler industry, these data can provide fresh insight into the molecular processes underlying the regulation of fat deposition in broilers.

It is concluded that hawthorn extract may improve the weight gain and FCR and decrease the abdominal fat in broiler chickens. Consumption of this extract can also attenuate oxidative stress through decreasing the lipid peroxidation and potentiate the total antioxidant capacity especially via up‐regulation of CAT, SOD1 GPX1 enzymes. On the other hand, hawthorn through improving lipid profile and reducing the expression of key lipogenic enzymes is capable of suppressing lipogenesis and imply its antihyperlipidemic effect. However, a few studies have been published about the effects of hawthorn on antioxidant and antihyperlipidemic conditions as well as antioxidant and lipogenic gene expression in the liver of broiler chickens. So, further systematic in vivo and in vitro research is warranted to explore and verify the potential effect of hawthorn extract to provide precise guidance for clinical use and new drug discovery.

## AUTHOR CONTRIBUTIONS

Behnam Ahmadipour conceived and designed research and conducted experiments. Samira Abaszadeh, Behnam Ahmadipour, Majid Kalantar and Hossein Hassanpour contributed to the collection of samples and the data. Behnam Ahmadipour and Majid Kalantar analysed the data. Behnam Ahmadipour, Samira Abaszadeh, Majid Kalantar and Hossein Hassanpour wrote the manuscript. All the authors read and approved the final manuscript.

## CONFLICT OF INTEREST STATEMENT

The authors declare there is no conflicts of interest.

## FUNDING INFORMATION

Shahrekord University

## ETHICS STATEMENT

The Animal Care and Use Committee of Shahrekord University approved all of the animal welfare and experimental protocols used in the study (SKU‐1402R).

## Data Availability

Data are ready upon request.
